# Changes of gonadotropin-releasing hormone receptor 2 during the anadromous spawning migration in *Coilia nasus*

**DOI:** 10.1186/s12861-016-0142-9

**Published:** 2016-11-24

**Authors:** Jin-Rong Duan, Di-An Fang, Min-Ying Zhang, Kai Liu, Yan-Feng Zhou, Dong-Po Xu, Pao Xu, Da-Peng Li

**Affiliations:** 1 Freshwater Fisheries Research Center, Chinese Academy of Fishery Sciences, 9 Shanshui Road, Wuxi, 214128 China; 2 Key Laboratory of Freshwater Animal Breeding, Ministry of Agriculture, Huazhong Agricultural University, Wuhan, 430070 China

**Keywords:** GnRH-R2, Spawning upstream migration, *Coilia nasus*, Yangtze River

## Abstract

**Background:**

An increase in the activity of the pituitary-gonad axis (PG-axis) and gonad development are essential for the onset of spawning migration in teleosts. In the fish *Coilia nasus*, gonad development and spawning migration up the Yangtze River occurs by the end of each summer. We hypothesized that gonadotropin releasing hormones receptor 2 (GnRH-R2), which together produce a signal that interacts with the PG-axis, may help to regulate spawning migration processes.

**Results:**

In this regard, we (1) characterized the gonadosomatic index (GSI) in the anadromous fish *C. nasus*; (2) analyzed the GnRH-R2 mRNA expression levels in ovary and brain, and concentrations in the serum; and (3) identified the GnRH-R2 protein distribution in the brain and ovaries. We found strong relationships between all of these indices.

**Conclusions:**

The results indicate that GnRH-R2 could act together to promote spawning during the anadromous migration. There is some evidence that the GnRH-R2 gene expression levels and protein distributions change in association with the migratory behavior.

## Background

Gonad maturation and spawning migration in teleosts are primarily regulated by pituitary–gonad axis (PG) neurohormones [1, 2]. The hypothalamic neutrohormone, gonadotropin-releasing hormone (GnRH), helps to regulate gonad function and spawning migration behaviors in fish [3–6]. GnRH stimulates the synthesis and release of pituitary gonadotropins, including follicle stimulating hormone and luteinizing hormone; these hormones then stimulate spawning behavior [7, 8]. Analyses of how GnRH is involved in spawning processes are complicated, because there are multiple forms of GnRH in the brains of some fish species [9–11]. In association with the different forms of GnRH, there are multiple forms of GnRH receptors (GnRH-Rs); these receptors bind to GnRH, initiating the intracellular signaling system [9, 12]. GnRHs and GnRH-Rs have also been found in gonads of fish [13, 14].

GnRH-Rs were first cloned from mouse pituitary cells [15, 16]. The first non-mammalian GnRH-Rs were obtained from African catfish [17]. However, even though nearly 30 years have passed, in fish species, the distribution of GnRH-Rs in cells and tissues, their regulation, and their functions remain elusive. By characterizing GnRH-R expression levels, we could improve understanding of the physiological consequences of GnRH stimulation [18]. In addition, the characterization of GnRH-R gene expression in a single species would help to clarify the mechanisms that regulate GnRH functions [19]. Most studies on GnRH-Rs have focused on the pituitary gland and few have considered the gonads. For example, the stimulation of GnRH both up- and down-regulated the GnRH-Rs in the pituitary gland [12]. Thus, little is known about the regulation of GnRH-Rs in the gonads.

*Coilia nasus* (Clupeiformes: Engraulidae), the Japanese grenadier anchovy, is a small-moderate sized fish [20–22]. *C. nasus* is an anadromous species; every year it migrates from the sea up to the middle and lower reaches of rivers in China, including the Yangtze River, and the lakes connected to it [21, 23]. *C. nasus* reaches sexual maturity at 2–3 years old. It lays eggs from March to July, breeding once a year [24]. *C. nasus* provides a classic case-study for yearly spawning behavior, with migration distances that reach thousands of miles [20, 25].

Changes in the aquatic ecology of the Yangtze River almost caused extinction of C. nasus in its middle reaches [26, 27]. As a result, attempts have been made to alleviate the threat to *C. nasus* resources [28]. Several research projects into artificial breeding and larval rearing techniques have been done [28, 29]. However, these studies have been limited to the biological characteristics and genetic diversity of the species [30–33]. The endocrine mechanisms involved in regulating migration and spawning in *C. nasus* have not been considered, to our knowledge.

To investigate the role of GnRH-Rs play in regulating fish spawning migration and gonadal maturation, we analyzed changes in the gonadosomatic index (GSI %) and used enzyme-linked immunosorbent assays (ELISAs) to test serum concentrations of GnRH-R2 during different spawning stages. Furthermore, we cloned the GnRH-R2 gene in *C. nasus* and examined their expression patterns in the brain and ovary using real-time quantitative PCR (RTqPCR). The GnRH-R2 protein distributions were also identified, in both the brain and ovary, using immunohistochemistry (IHC). The present study enabled us to understand the function of GnRH-R2 in *C. nasus*, in association with the onset and development of spawning migration in the species.

## Methods

### Fish sampling

**Fig. 1:**
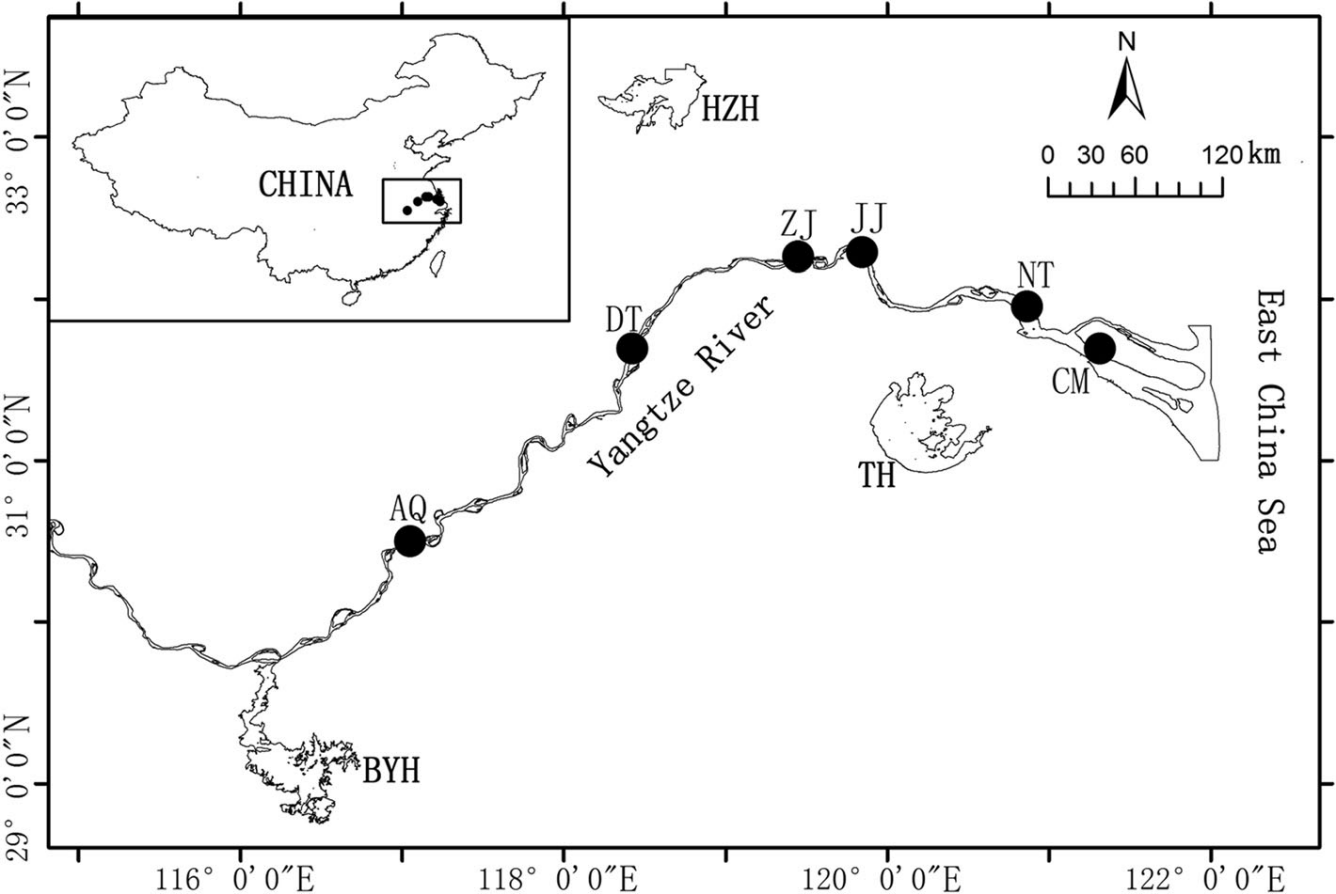
Sampling distribution locations for *C. nasus* in the Yangze River. Black dot display the sampling distribution locations. AQ: Anqing; DT: Dangtu; ZJ: Zhenjiang; JJ: Jingjiang; NT: Nantong; CM: Chongming, TH: Taihu lake; HZH: hongzehu lake; BYH: Boyanghu lake

In 2015, from March to August, healthy fish (*n* = 98) from six populations of *C. nasus*, were collected at seven time points from their major regional habitats in the Yangtze River during their migration upstream (Fig. 1). The collection sites were at: Anqing (AQ); Dangtu (DT); Zhenjiang (ZJ); Jingjiang (JJ); Nantong (NT), and Chongming (CM). Fish were kept on dry ice immediately after collection, and were transferred to the laboratory in the dry ice boxes.

### Analysis of development stage and tissue collection

After measuring the body weights (BW ± 0.01 g wet weight; WW) of the fish their gonads were dissected and their genders were determined; only female fish (total 98 individuals,14 fish per time point) were studied for GSI analysis. The gonad weight (GW ± 0.01 g WW) were recorded so that the GSI of each fish could be calculated (GSI = GW/BW × 100) for each population (mean ± standard deviation).

Based on visual judgment of the gonads and microscopic examination of the oocytes, each female was assigned to one of the following seven stages of oocyte development [34]: onset stage (stage I; fish collected in March), developmental stage (stage II; April), multiplication stage (stage III; May), mature stage (stage IV; June), spawning stage (stage V; late June), spawned stage (stage VI; July), or rest stage (stage VII; August). All fish experimental procedures were performed according to the Regulations for the Administration of Affairs Concerning Experimental Animals approved and authorized by the State Council of People’s Republic of China.

### RNA extraction

During dissection, the brain and ovary tissues from individuals at each oocyte development stage (*n*=5) were selected randomly for RNA extraction and the extracts were pooled to form one sample for mRNA expression analysis. Total RNA was extracted using Trizol Lysis Reagent and then purified with an RNA easy kit (Invitrogen, Beijing, China), according to the manufacturer’s instructions. The RNA integrity and quantity were estimated by spectrophotometry (absorbance at 260 nm) and agarose gel electrophoresis, respectively.

### RTqPCR analysis

To analyze the mRNA transcript expression patterns at each fish developmental stage, total RNA (about 2 μg) that had been isolated from the brains and ovary tissues was reverse transcribed into cDNA using the SMART^TM^ cDNA kit (Clonetech, USA) and RTqPCR analysis was performed, using the PrimeScript Real-time PCR Kit (TaKaRa, Japan). Target fragments of cDNA that encode GnRH-R2 were chosen basing on the constructed transcriptome library using BLAST tool [35]. First-strand cDNA was prepared as described above; the gene-specific primer pair (GnRH-R2-F and GnRH-R2-R; Table 1) were designed based on the cDNA sequences (GenBank accession numbers KU861569) to produce 387 bp amplicons. The PCR reaction conditions were as per the qPCR Kit protocol. Samples were run in triplicate using pooled RNA (as described above) at the same concentration, and normalized to the selected control gene18sRNA; the primer pair 18sRNA-R and 18sRNA-F (Table 1) were designed based on the *C. nasus* 18sRNA and to amplify a fragment of 232 bp. The gene expression levels were calculated using the 2^−ΔΔCt^ comparative CT method [36]. Mean and standard deviation values were calculated from the triplicate runs, and presented as fold differences in expression, relative to 18 s RNA expression. Data were analyzed using CFX Manager^TM^ software (version 1.0).

**Table 1:** Sequences of primers used in the present study

Primer Name F—Forward/R—Reverse	DNA-Sequence 5′-3′	Annealing Temperature (°C)	Fragment Size (bp)
Gene-specific Primer pairs for RT-qPCR			
GnRH-R2-F	5′-CGTGCGGTGAAGGCGAAGGGGGTGG-3′	60.7	387
GnRH-R2-R	5′-ACACAACCCCAACTAAGCAAGCATA-3′	64.7	
18sRNA primers			
18sRNA-R	5′- TGATTGGGACTGGGGATTGAA-3′	59.2	232
18sRNA-F	5′- TAGCGACGGGCGGTGTGT-3′	62.4	

### GnRH-R2 antiserum preparing

GnRH-R2 antiserum was produced commercially by Hua-an Biol. Co., Ltd. (Hua-an, Hangzhou, China). Briefly, a synthetic signature peptide (LVVVSLDRH) for GnRH-R2, conjugated with the keyhole limpet hemocyanin, was emulsified with complete (for the first immunization) and incomplete (for the second to fourth immunizations) Freund’s adjuvant, and injected into a New Zealand rabbit at intervals of 2 to 3 weeks. Before immunization and after the third and fourth injections, the rabbit was bled and serum samples were collected. An increase in antibody titers against the peptide was verified by enzyme-linked immuno sorbent assay (ELISA).

### ELISA

ELISAs were used to measure the GnRH-R2 concentrations in the fish serum. Re-GnRH-R2 were diluted in 50 mM carbonate buffer (pH 9.6), to produce concentrations of 30 ng/mL and 40 ng/mL, respectively. Ninety-six well polystyrene plates were coated with 50 μL per well of re-GnRH-R2 solution overnight at 4 °C. The wells were then washed three times with phosphate-buffered saline (PBS) containing 0.05 % Tween20 (TPBS). Then, 100 μL of Superblock in PBS was put into each well for 1 h at room temperature (RT) before the antigen-coated plate was washed with PBS again.

Serum samples (*n*=6 for each development stage) from *C. nasus* were diluted 1:8 with TPBS. TPBS with 5 % goat serum was used to dilute the primary antibodies (anti-GnRH-R2) at a ratio of 150:1. In 1.5 mL microtubes, each sample and standard was mixed with equal amounts of each primary antibody (separately). 50 μL of each reaction mixture was dispensed into separate wells on the antigen-coated plate in triplicate. The plate was incubated at 4 °C overnight.

After incubation, 50 μL of solution containing biotinylated antibody to rabbit immunoglobulins (Zymed, CA, USA) diluted at a ratio of 1:10 in 5 % NGS-TPBS, was allotted to each well for 1.5 h at RT. The wells were then washed and further incubated, for 1 h at RT with 50 μL (per well) of streptavidin-polyHRP80 (Fitzgerald, CA, USA) diluted into 200 ng/mL with Universal Casein Diluent/Blocker. The wells were washed again and then 100 μL of 1-Step Ultra TMB-ELISA solution (Thermo Scientific, Waltham, USA) was dispensed into each well for development for 30–60 min at RT. The reaction was stopped by adding 100 μL of 2 M sulfuric acid. Absorbance was read at 450 nm.

The optical density results of the pooled serum samples’ serial dilutions and those of the standards were used to validate the serum GnRH-R2 levels. The optical densities of related substances (including glycoprotein hormones and their subunits) were compared with those of the standards to analyze the specificity of the experiments. The precision of the assays was assessed from the intra-and inter-assay coefficients of variation (CV) from the same samples. The concentration of GnRH-R2 in the samples was calculated from the standard curve, constructed using the optical densities of the standards in ELISA analysis software (Magellan, Tecan, Männedorf).

### IHC

During dissection, sexually mature individuals (*n* = 3, stage IV) were selected for the IHC experiment. Ovaries were fixed in 0.01 M PBS containing 4 % paraformaldehyde and stored at 4 °C overnight. After washing with PBS three times, ovaries were dehydrated in 20 % saccharose-PBS solution for 4 h at RT. Then they were embedded in organ optimal cutting temperature compound (Sakure, CA, USA). Standard frozen sections (8 μm in thickness) were taken using a microtome (Leica, Bensheim, Germany). Then, after washing with 0.01 M PBS three times (each wash, 10 min), the sections were immersed in 0.01 M citric acid buffer (pH 6.0) containing 0.1 % Tween 20, and autoclaved for 5 min. Following this, a blocking solution (Roche, Shanghai, China) was used to treat the sections. Anti-GnRH-R2 (1:200) was added to the sections and then they were incubated overnight at 4 °C, then rinsed with 0.01 M PBS three times (each wash, 5 min). Then goat anti-rabbit IgG conjugated with horseradish peroxidase were added and the sections were incubated for 30 min, before being rinsed with PBS three times (each wash, 5 min). Diaminobenzidine (Sigma, Shanghai, China) was used as the substrate to visualize the immunoreactive signals and the sections were counterstained with H & E. As a negative control, organ sections were also incubated with pre-immune rabbit serum and blocking solution.

### Statistical analysis

A multiple comparisons (Duncan’s) test was used to compare differences in GSI, GnRH-R2 concentrations in the serum, and gene expression in the ovary and brain tissue, among the control and tested samples (*P* < 0.05).

## Results

**Fig. 2:**
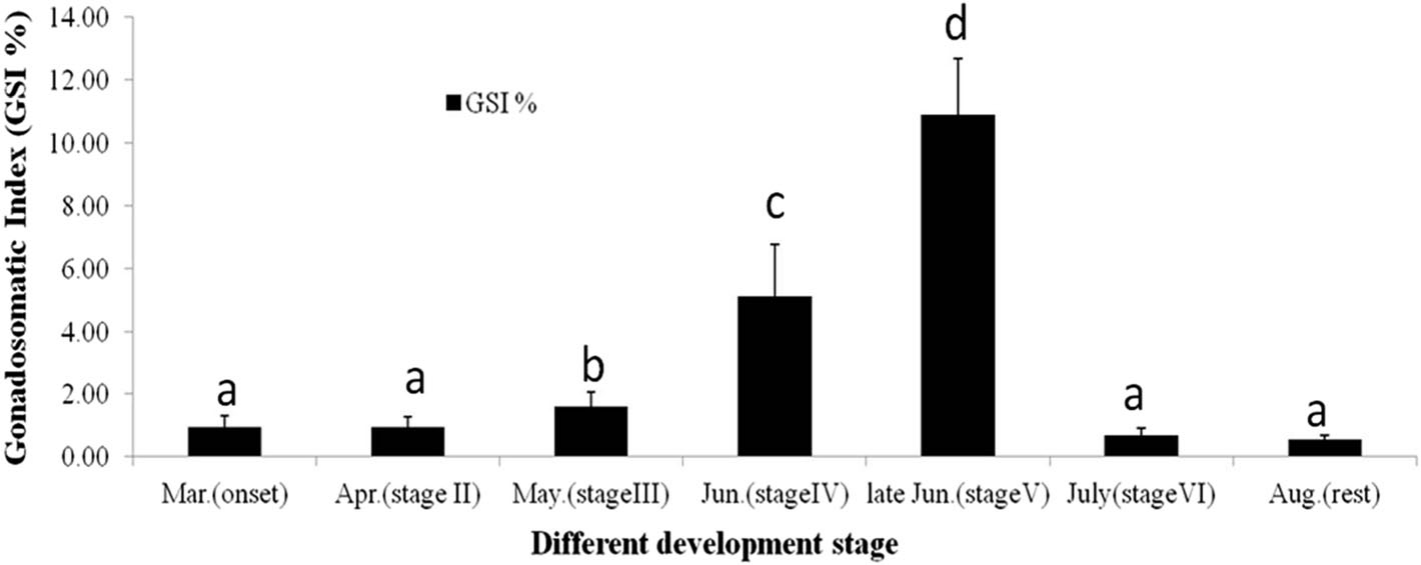
Changes in the gonadosomatic index (GSI) during the oocyte development stages in *Coilia nasus*. The stages were defined as: onset stage (stage I; fish collected in March), developmental stage (stage II; April), multiplication stage (stage III; May), mature stage (stage IV; June), spawning stage (stage V; late June), spawned stage (stage VI; July), and rest stage (stage VII; August). Data are expressed as the mean ± SE (*n* = 7 for each stage). Columns with different letters above them are significantly different (*P* < 0.05, a<b<c <d)

In total, 98 *C. nasus* female fish (14 per time point) were sampled from the onset migration stage to the rest stage (from March to August, 2015) for the GSI analysis (Fig. 2). During March and April (stages I and II), the female fish GSI was very low (<1.0 %, Fig. 2). In May (stage III), the GSI increased slightly, but significantly (GSI = 1.59 %); in June (stage IV), the female GSI increased sharply (GSI = 5.12, *P* < 0.05) and this increase continued into late June (stage V), when the highest GSI was recorded, during the spawning stage (GSI = 10.09 %). After the spawning stage (in July, stage VI), the GSI decreased sharply in the spawned stage (GSI = 0.71 %) and was at very low levels, similar to those in March and April, for the rest stage in August (GSI = 0.65 %).

**Fig. 3:**
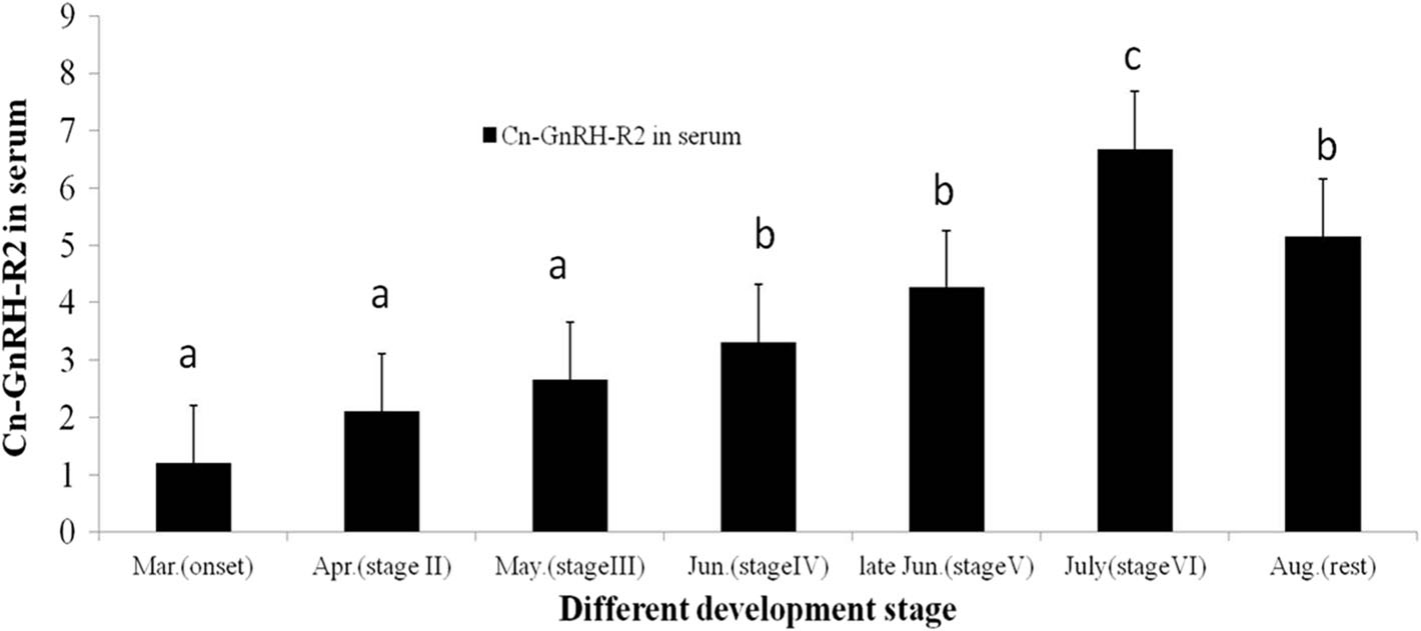
GnRH-R2 concentration in serum during the oocyte development stage in *Coila nasus*. The stages were defined as: onset stage (stage I; fish collected in March), developmental stage (stage II; April), multiplication stage (stage III; May), mature stage (stage IV; June), spawning stage (stage V; late June), spawned stage (stage VI; July), and rest stage (stage VII; August). Data were collected by enzyme-linked immunosorbent assays and are expressed as the mean ± SE (*n* = 3). Columns with different letters above them are significantly different (*P* < 0.05, a<b<c)

The GnRH-R2 concentration in the serum during the migration stages of the fish changed interestedly (Fig. 3). There was a gradual increase in the serum GnRH-R2 concentrations from the onset (stage I) to multiplication (stage III), and then a sharp increase to the highest concentration of GnRH-R2 observed during maturation (stage IV); after the mature stage, significantly high levels (though not as high as in stage IV) of GnRH-R2 were maintained during the spawning and spawned stages (stage V and VI), and then there was a sharp decrease for the rest stage.

**Fig. 4:**
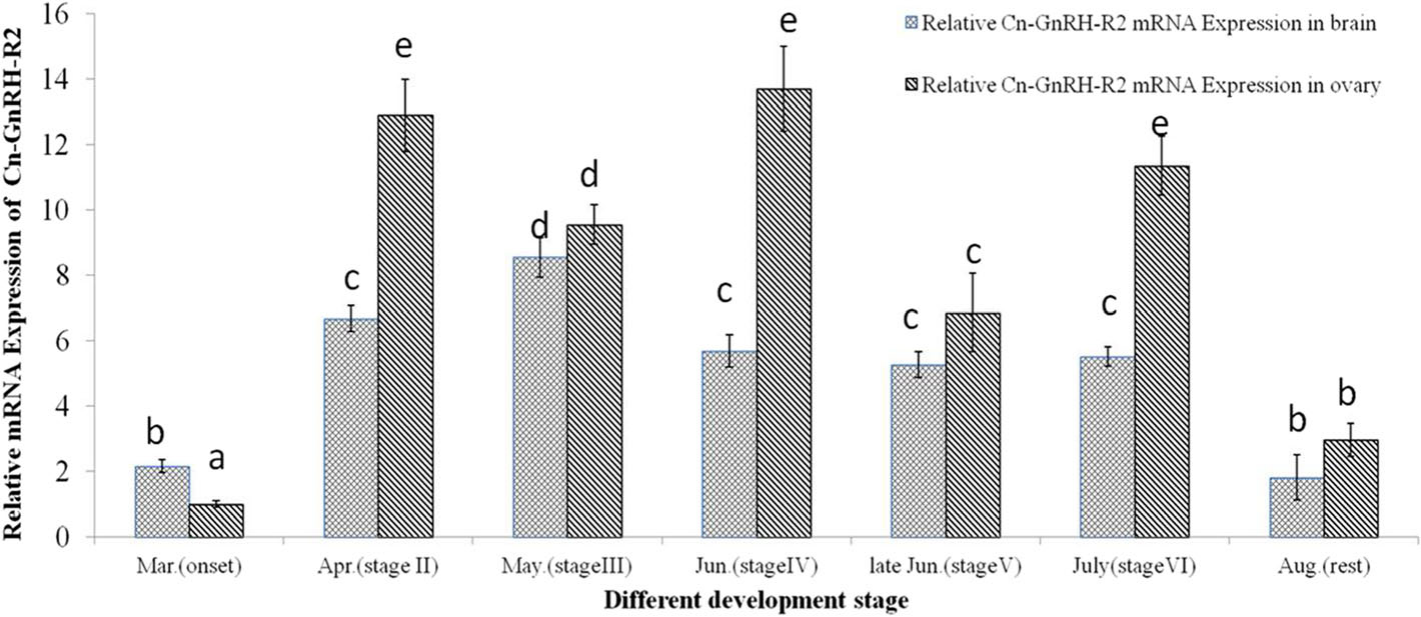
Changes in expression of GnRH-R2 mRNA in brain and ovary tissues during the oocyte development stage in *Coila nasus*. The stages were defined as above. Expression values were normalized to those of 18sRNA. Data are expressed as the mean fold difference (mean ± SE, *n* = 3) compared to the 18sRNA expression. Columns with the different letters above them are significantly different (*P* < 0.05, a<b<c <d<e)

The temporal expression levels of the GnRH-R2 mRNA transcripts in the brain and ovary during the migration cycle are presented in Fig. 4. The GnRH-R2 mRNA transcription (Fig. 4) were maintained high levels during the migration stages (from stage II in April to stage VI in July) in both the brain and ovaries. Interestingly, three peaks in GnRH-R2 mRNA transcription within the ovaries were observed during that time period: one in the developmental stage (stage II; April), the second in the maturation stage (stage IV; June) and the third peak was in the spawned stage (stage VI; July). GnRH-R2 mRNA transcription in the brain peaked in the multiplication stage (stage III; May). Higher expression levels (*P* < 0.05) were detected in the ovary from the developmental stage to the rest stage (April–August).

**Fig. 5:**
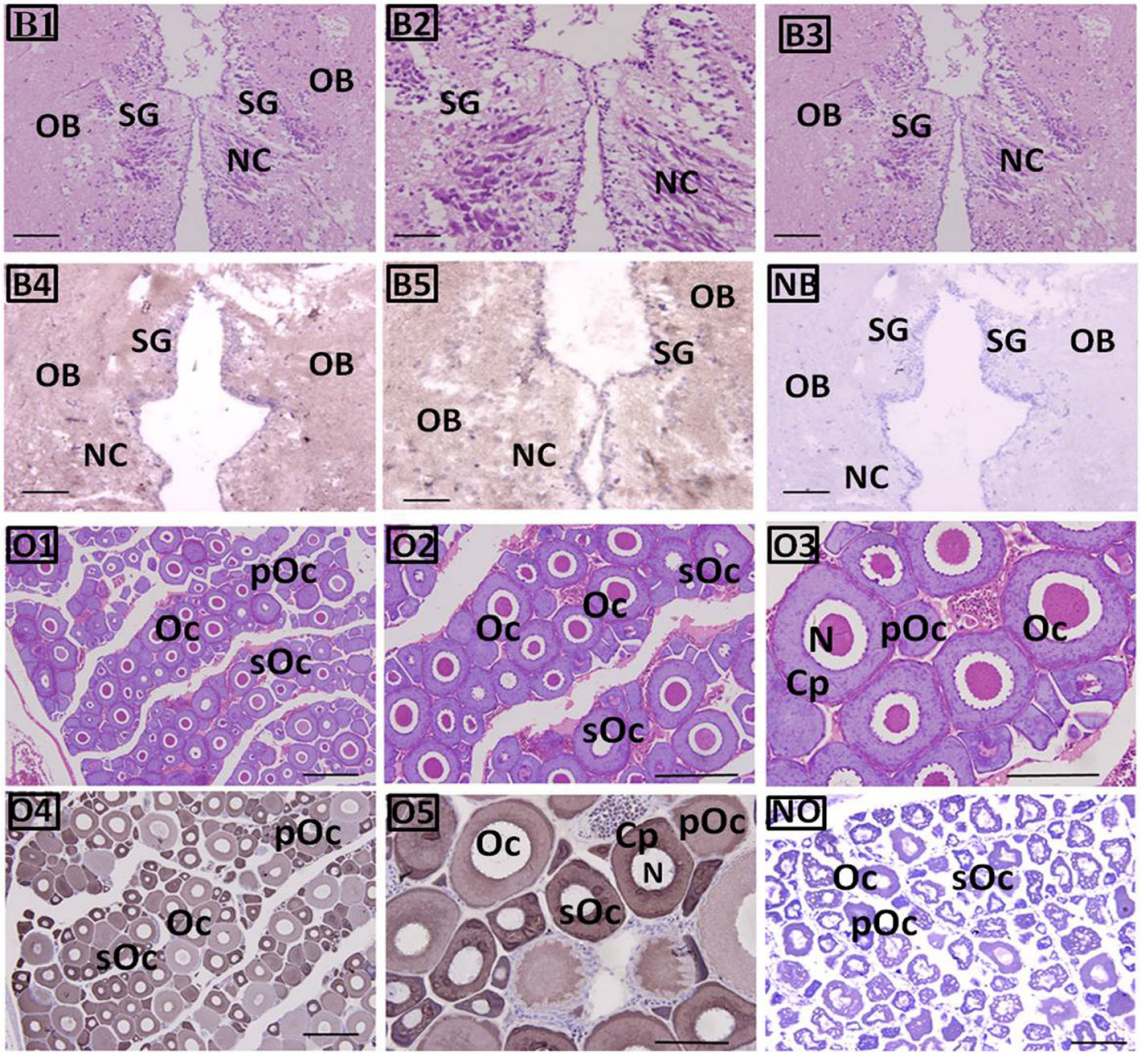
GnRH-R2 locations in the brain and ovary tissues. Immunohistochemical positive signals of GnRH-R2 immunolabeling are shown in brown. B1–B3: whole brain sections stained with H&E; B4–B5: GnRH-R2 immunolabels in different parts of the brain; and NB: negative control for brain. O1–O3: whole ovary sections stained with H&E; O4–O5: GnRH-R2 immunolabels in different parts and oocyte developmental stages of the ovary for IHC (O5 provides a more magnified view of the ovary IHC signal); and NO: negative control for the ovary tissue. Key: OB, olfactory bulb; SG, stratum granulare; NC, Neurogliocyte, pOc, primary oocyte; sOc, secondary oocyte; Oc, oocyte; N, oocyte nucleus; and Cp, oocyte cytoplasm. Scale bar = 100 μm

The whole section of the brain and ovary, stained with hematoxylin-eosin (H & E) and with anti-GnRH-R2 immunolabeling (counterstained with H & E), from the IHC analysis, are shown in Fig. 5. Immunoreactive positive signals for the GnRH-R2 protein were detected; in the brain and ovary tissues, the positive signals for GnRH-R2 were mainly found in the cytoplasm of olfactory bulb (OB) cells, stratum granulare (SG) cells and in oocytes at different developmental stages. The strongest signals were found in the primary oocytes; lower positive signals were found in the cytoplasm of OB and SG cells; and weak signals or an absence of any signal were observed in the mature oocytes and neurogliocyte cells. No positive signals were observed in the negative control, which was incubated with the pre-immune rabbit serum (Fig. 5).

## Discussion

There were significant differences in the GSI among the different oocyte developmental stages of the anadromous female fish *C. nasus*. Usually, in late March *C. nasus* begins spawning, moving upstream between the Chongming and Anqing sections of the Yangtze River, and then the fish enter into their resting stage [24]. After a short time of recruitment, the fish then begin to migrate downstream and into the sea, finishing the migration spawning cycle [34]. The females’ GSI levels were very low in the onset and development stages; these fish were mainly recruiting and their gonads were just beginning to start development. From May to early July, the GSI levels significantly increased, as the fish spawned in the upstream reaches of the river. During the spawning stage, when the fish were located upstream, ovaries were physically stimulated for further development, growing bigger and the GSI increasing, accordingly, until the fish had spawned.

To investigate the trigger of ovary maturation and spawning migration in *C. nasus*, we measured the serum GnRH-R2 concentration. The serum GnRH-R2 concentration was at its maximum in June (mature stage) and was maintained at high concentration during late June (spawning stage) and when they had just spawned in July. Similar in the protandrous black porgy fish, *Acanthopagrus schlegeli*, the expression profiles of both forms of GnRH-R were variable in the gonads according to the gonadal stage and season [13]. Combing with our findings for *C. nasus*, the results indicated that the high GnRH-R2 level may have been a biological response to migration/spawning behavior [13, 37]. Furthermore, the high level of GnRH-R2 in the serum during the different stages of oocyte development could demonstrate their crucial role in regulating the spawning migration.

During the migration period, the GnRH-R2 mRNA transcripts were preferentially expressed in the brain and ovary tissues; mRNA expression levels were closely related to the different migration stages. The GnRH-R2 mRNA expression levels in the brain were significantly high, which indicates that it would play a dominant role in regulating migration/spawning behavior [37]. GnRH-R2 mRNA expression levels significantly increased over time during the *C. nasus* migration cycle, which may be related to maintaining high GnRH mRNA levels during the mature oocyte stage. Overall the GnRH-R2 mRNA expression levels changed quite similarly during the spawning period, indicating that GnRH-R2 would be closely involved in ovary maturation and migration behavior in *C. nasus*. The mRNA expression patterns at the different oocyte development stages in *C. nasus* were inconsistent with those observed in farmed salmon [11], the amounts of GnRH-R2 mRNA in the forebrain, were high between winter and spring, in the prepubertal stages, then declined as summer approached, and finally increased again in the spawning season [38]. According to the salmon, since the maturing *C. nasus* leave the East Sea for the egg laying sites in the Yangtze River, gene expression for GnRH-R2 mRNA should have been high and should have accumulated for several months (from March to May), prior to the mature oocyte stage. Interestingly, the GnRH-R2 mRNA transcripts exhibited three peaks in the ovary (Fig. 4) indicating that GnRH-R2 may be closely related to oocyte development and maturation, and post-spawning. In *Odontesthes bonariensis*, several subtypes of GnRH receptors in the pituitary also had transient peaks in concentration from January to March [38, 39]. In contrast, in masu salmon, the amounts of GnRH peptide in the pituitary gradually increased with gonad maturation, from spring to autumn [39, 40]. This would then have promoted fish to migrate from the estuary up to the Yangtze River from March to July.

The mechanism by which GnRH-R2 regulates the spawning migration is still unclear and further research is needed. sGnRH neurons have been shown to help control the PG-axis in maturing adult salmon in the summer [41]. Therefore, sGnRH gene expression is likely to increase before any elevations in the PG-axis activity of chum salmon in the summer, in the Bering Sea [38]. The present study shows that gene expression of GnRH-R2 was elevated in association with the activation of the PG-axis, during the upstream migration of *C. nasus* (Fig. 4). These results support the seasonal profiles of GnRH-R2 mRNA levels in *C. nasus* are likely to be similar to sGnRH change patterns found in chum salmon [38].

Several studies have measured brain or ovary GnRH-R2 proteins by IHC, during gonad maturation [18]. It is thought that only one form of GnRH-R2, with neurons located in the preoptic area, regulates secretion of gonadotropins in the pituitary, although two or three forms of the GnRH molecule can exist within the same species [42]. The IHC results of the present study revealed higher expression levels of GnRH-R2 in the olfactory bulb upper cells, stratum granular cells and early stage germ cells, including the primary oocytes, in abundance from the beginning of spawning migration [37]. There was weak or no expression in neurogliocytes and mature oocytes. These findings are supported by data from other species and indicate that the GnRH-R2 protein is mainly needed at the onset of migration behavior [37]. The GnRH-R2 protein was also found abundant in the cytoplasm of the germ cells during their developmental and multiplication stages; this suggests that the cytoplasmic protein assembling machinery, through which additional proteins needed for cell division are generated, is very active during those stages. In this sense, it is likely that GnRH-R2 modulates spawning migration in *C. nasus* by both regulating cytoplasmic organization in the germ cells and stimulating the synthesis and release of gonad hormones during the different spawning stages.

## Conclusions

The present study provided evidence that the GnRH-R2 gene expression and protein distribution in the brain and ovaries changed in association with the migratory behavior. The results indicated that GnRH-R2 could act as a mediator; promoting spawning behavior in the anadromous teleost fish *C. nasus*. However, the present study was conducted under natural spawning conditions. The photoperiod and/or water temperature changes could stimulate or delay the maturation of *C. nasus* ovaries. Thus, further study of the influence of environmental factors that regulate gonad maturation and spawning migration in *C. nasus* are needed. To conclude that GnRH-R2 are functionally involved in gonadal maturation or spawning behavior in the *C. nasus*, it is necessary to comprehensively examine the effects of GnRH-R2 on the PG-axis neurohormones synthesis and release both in vivo and in vitro.

## Abbreviations

BW: Body weights
CV: Coefficients of variation
ELISA: Enzyme-linked immuno sorbent assay
GnRH: Gonadotropin-releasing hormone
GnRH-R2: Gonadotropin releasing hormones receptor 2
GSI: The gonadosomatic index
H&E: Hematoxylin and Eeosin
IHC: Immunohistochemistry
OB: Olfactory bulb
PBS: Phosphate-buffered saline
PG: Pituitary–gonad axis
RT: Room temperature
SG: Stratum granulare
WW: Wet weight
